# Liston and Dieffenbach

**Published:** 1848-02

**Authors:** 


*Liston and Dieffenbach.*—Within four weeks of each other we have to deplore the deaths of Liston and Dieffenbach, both of whom acquired a degree of professional renown that entitles them to consideration in our pages.

Robert Liston was born on the 28th of October, 1794, in the Manse of Ecclesmachan, Linlithgow. His father, the Rev. Henry Liston, was the minister of that parish, and distinguished for his musical acquirements and classical knowledge. Under his tuition Robert became a sound Latin scholar; so that, on subsequently attending the humanity classes in Edinburgh, he obtained a prize for composition in that language. In 1810, he commenced the study of medicine by attending the lectures of Dr. Barclay, who afterwards appointed him his assistant, in which capacity he distinguished himself as a skilful anatomist. During the subsequent six years he followed the usual course of medical education, and went through the office of dresser, clinical clerk, and house clerk in the Royal Infirmary. In 1816 he visited London, where he studied a year under Sir William Blizard, and Mr. Thomas Blizard, of the London Hospital, and Mr. Abernethy at St. Bartholomews. He became a member of the Royal College of Surgeons in London, and subsequently returned to Edinburgh, where he established himself. He was elected a Fellow of the Royal College of Surgeons of that city in 1818, writing, for his probationary essay, "On Strictures of the Urethra, and some of their consequences."

Mr. Liston now commenced his career as a lecturer on anatomy and surgery, and early became remarkable for the boldness and skill with which he operated. At that time surgery was not so advanced in Edinburgh as it afterwards became; and many cases considered incurable by the officers of the Royal Infirmary, fell into his hands on being discharged from that institution, and were by him made subjects of highly successful operations. His rivals charged him with inveigling patients out of the Infirmary for that purpose, a charge which he denied, boldly asserting his right to operate on and benefit those who had been considered incurable by others. One case especially excited great attention. It was that of a tumour firmly attached to the scapula; which, after a consultation of the surgeons of the Infirmary, was considered an improper subject for operation, and discharged. Mr. Liston afterwards resolved on removing it, together with a part of the shoulder blade, an operation which he performed on the 16th of November, 1819. Unfortunately the disease returned in the portion of the bone which was left, just as the wound was well; when Mr. Liston proposed the removal of the diseased bone and arm as the only means of saving life. No surgeon in Edinburgh, however, would assist him in the operation, and he was obliged to abandon the poor boy to his fate. He died three months after, and the surgeon in Kinross who examined the body, on forwarding to him the diseased part, stated, "We performed, in its removal precisely the operation which you proposed to save the unfortunate lad; and I now regret that it was not done when he was last under your care. The disease, you will perceive, had no connexion with any of the vital organs."

This, and several other cases of a similar kind, gave rise to great discussion in the Infirmary, and it was proposed to him on the part of the managers, that he should refuse his assistance to any person who had been a patient in that institution, and that he should abstain from visiting or attending the wards. He declined both these propositions, and, in consequence, was expelled, and never entered its gates for a period of five years. At the expiration of that time, however, in

1827, he was suddenly elected one of the surgeons, not owing to any merit he possessed as an operator, but to some private influence which he skilfully forced to be exerted in his favour.

There was now opened to him a field for the proper display of his powers, and the boldness, skill, and rapidity of his operations acquired for him a European reputation. Who that once saw Liston perform a capital operation could ever forget it? The manner in which he handled the knife had in it something remarkable. It at once put the spectators at their ease, and convinced them that the instrument would not go any where, or do any thing, but what it ought. He possessed a confidence and self-possession that no untoward event ever disturbed; a readiness in suddenly applying expedients, that seemed to the inexperienced the result of fore-thought; and a power in his muscular arm and bony hands that enabled him to twist, depress, or elevate parts, with an ease that appeared almost magical. As a lithotomist he has never been surpassed, and his flap operations were performed with such rapidity, that the sound of sawing seemed to succeed immediately the first flash of the knife. With him the length of operations were estimated by seconds instead of minutes, and the student who unwarily turned his head to address his neighbour, on looking round discovered that what he had struggled so much to witness, had already been performed. In all this, however, there was no real hurry; it was the result of a skilful combination of movements, performed with precision and exactitude. In more complicated operations, when he wished to use both hands, he saved much time, holding the knife by the handle in his mouth, instead of entrusting it to an assistant, or laying it down, a habit he was led to adopt from having carefully watched the manner in which the butchers of Edinburgh rapidly separated the different joints of meat.

As an example of the readiness with which he encountered difficulties, we may relate the following incident, communicated to us by one who was present. He had amputated a leg for extensive necrosis and chronic disease of its bones. The patient was feeble, and yet, after securing many vessels, hemorrhage was still abundant from the cut surfaces. More vessels were secured, but the bleeding continued, and Professor Russel, who was assisting him, became alarmed lest the patient should sink on the table. At this moment it was discovered that the blood oozed from an enlarged vessel in the substance of the bone. There was a panic of a few seconds, and every one whispered, what was to be done? Liston immediately sliced off a piece of wood from the operating table with his large amputating knife, and rapidly formed it into a plug, which he thrust into the mouth of the bleeding vessel. The hemorrhage ceased immediately, and the patient recovered.

In 1835, he accepted the invitation of the Council of University College to fill the chair of Clinical Surgery, and left Edinburgh for London. He soon obtained a large practice, and, on the death of Sir Anthony Carlisle, in 1840, was appointed member of the council of the college of surgeons. In 1846, he became one of the board of examiners, and had thus reached almost the highest honours of the profession in the capital of the empire, when his life terminated.

In the spring of 1847, he complained of constriction in the larynx, a sense of choking on stooping forwards, and difficulty of deglutition. Late in July, he coughed up between thirty and forty ounces of blood. He himself hinted at the possibility of an aneurism existing; but there were no physical signs of such a lesion perceptible to his medical attendants. In the beginning of October, the cough returned. became gradually worse, and was attended with rusty-coloured sputa. On the 1st of December, he was seized with what appeared to be spasmodic asthma. Attacks of dyspnœa supervened, which, during the six following days, gradually became more frequent and intense, and produced such exhaustion that he died on the evening of the 7th. On examination, an aneurism of the arch of the aorta was discovered, pressing upon the trachea, the mucous membrane of which was perforated by ulceration in three or four places, although the apertures were blocked up by the coagula in the sac of the aneurism.

Mr. Liston's contributions to the literature of his profession were not very numerous. His first publication is entitled, "Memoir on the Formation and Connexions of the Crural Arch, and other parts concerned in Inguinal and Femoral Hernia, 4to. 1819, with three plates." He communicated, also, to the "Edinburgh Medical and Surgical Journal" some papers, the first of which was inserted in the 16th volume of that periodical, and consisted of cases of aneurism, and an account of a case of fracture of the neck of the femur, in which a bony reunion had taken place within the capsular ligament. Other later Memoirs were inserted in the Transactions of the Royal Medico-Chirurgical Society of London. In 1831, he published his "Elements of Surgery," and, in 1837, the "Practical Surgery," a work which has gone through four editions, and is justly esteemed as one of the best guides on the subject of which it treats, now extant.

The improvements he introduced into the art of surgery, though few, were not unimportant. To him the surgical school of Edinburgh is indebted for the simple mode of dressing wounds which at present prevails, by lint saturated in water and covered with oil silk, instead of the greasy applications, strapping, and continued poulticing, which formerly prevailed. He was a great simplifier of instruments, his profound knowledge of anatomy enabling him to guide the straight bistoury with the utmost precision in the deepest and darkest recesses of the body. The bone nippers which he invented has every where been recognized as a most invaluable instrument to the surgeon, and, in his powerful hands, did wonders. We have seen him cut through the femur of a child with them at once, without any apparent effort. His operations also materially conduced to the introduction of the flap method of performing amputations, on which subject he wrote a paper. Such, we believe, are the principal claims he has upon the regards of posterity. The fact is, that celebrity with him did not so much depend on what he did, as on how it was performed. None possessed more practical tact, formed juster conclusions, and treated a case more appropriately, than Mr. Liston. All this he did intuitively, often without being enabled to give reasons for his mode of acting—a circumstance which caused him to make a better appearance at the bedside, and in the operating theatre, than in the lecture room. His medical treatment consisted principally in the exhibition of wine, antimony, and quinine, three remedies which he employed with consummate skill. We must not forget to mention that Mr. Liston has the merit of having early perceived the great advantages of investigating the minute structure of tissues and morbid growths by means of the microscope. On this subject he was quite *au courant* with the present state of science, and communicated to the Medico-Chirurgical Transactions of London an interesting paper on the vessels of lymph.

In private life Mr. Liston succceeded in forming many lasting friendships. It cannot be denied, however, that he frequently exhibited to those around him a degree of temper and coarseness of language which was often painful to witness. His apprentices, dressers, and clerks, however, got accustomed to this, and he had the art, by a subsequent degree of condescension, or a well-timed invitation to dinner, to make them forget his ill treatment. He was exceedingly fond of nautical pursuits, and of hunting. When in Edinburgh he frequently followed the hounds, and although a heavy weight, made a good appearance in the field. In London he kept a yacht on the Thames, in the relaxations of which he indulged up to a few weeks of his death. He was buried at the Highgate Cemetery, near which the hearse was met by upwards of four hundred of his pupils and his friends. These attended the body to the church, and from thence to the grave, where nearly three thousand persons, it is said, "were collected to pay their last testimony of respect to one whom, when living, they had been, in most instances, indebted for relief from personal suffering."

John Frederick Dieffenbach was a native of Königsburg, and a son of a professor of theology in the university of that city. Destined in early life for the church, Dieffenbach commenced his career as a student of theology, to the study of which, however, he seemed disinclined; for, at the age of eighteen, he volunteered into the Mecklenburg cavalry, and remained in that regiment during the last campaign for the deliverance of Germany. The war being ended, he resumed his theological studies, but soon devoted his talents to the study of surgery, under the tuition of Professor Walthus, in the university of Bonne.

In 1822, at the age of twenty-seven, he went to Paris and became the pupil of Larry and Dupuytren. His inaugural dissertation before the faculty of Wurzburg, on the transplantation of parts of the human body, seems to have been the first evidence of his partiality for that branch of surgery for which his name afterwards became so justly celebrated—rhinoplastic surgery.

On leaving Paris, he established himself in Berlin, where, from some interesting works which he published, and from his talents and extreme ingenuity as an operative surgeon, he very rapidly attracted notice. In 1830 he obtained a surgical appointment in the hospital of La Charité of Berlin, and at the death of Graefe in 1840, he succeeded that surgeon as clinical professor; in the hospital attached to which professorship lay the scene of most of his bold and ingenious operations, of his professional instructions, and lastly, of his sudden death.

On the day of his demise Dieffenbach appeared to be in the enjoyment of his usual vigorous health, and at his accustomed hour repaired to the hospital, where, seated on his sofa, he was conversing with two French physicians regarding a case of aneurism, on which he had operated the day previously. Suddenly he ceased speaking, his head fell forwards, and he died instantaneously.

The respect and veneration in which this great surgeon was held, was fully testified by the vast crowds which accompanied his remains to the place of burial.

The funeral procession, headed by a large volunteer band of musicians, consisted of the army surgeons, and many officers in full uniform, bearing the honours and decorations which had been conferred on the deceased; of the city clergy, the relations and private friends of the family; the rector and students of the university; the pupils of the Frederick Wilhelm Institution, and the medical men of Berlin. The state carriages of the King and Prince of Prussia, with a long line of private equipages, following the empty carriage of the deceased, closed the imposing and melancholy cortège. As the coffin was borne along through the streets, quantities of flowers and wreaths were showered over it by mourning citizens, who were assembled in vast numbers to pay this last touching tribute of respect to their distinguished surgeon.

In Dieffenbach the profession has lost a surgeon, whose fertile imagination, combined with neatness of execution and untiring zeal, has conferred on surgery much that has been of the greatest value; and although we have now to lament his being removed in the midst of his usefulness, still he has opened up paths in a new and till of late little explored branch of surgery, which will lead others to follow with increased zeal in his footsteps.

Admirable in all departments of surgery, Dieffenbach ever shone conspicuously in the surgical treatment of malformation, to which he devoted the greater part of his attention. He first performed the operations for the cure of squinting and stammering, and of the subcutaneous division of many muscles for the relief of deformities. The works which he has written, though elaborate, and containing much valuable practical matter, convey but an inadequate idea of his operative powers, and of his acute discernment in these, his favourite subjects of surgical treatment. He was remarkable for the ingenuity with which he planned and carried into execution many of the most difficult rhinoplastic operations. By those who have observed the precision with which he performed these operations; who have been eye-witnesses to the dexterity and coolness with which he would change his plan of execution in the middle of an operation, when others would have been embarrassed by the difficulties in which they had involved themselves; and by those who have watched the admirable results which have followed such operations in his hands, could Dieffenbach only be fully appreciated. No deformity ever baffled him. He delighted to restore the use of their limbs to those halt and maimed, whom others had given up as hopeless. The greater the amount of malformation, the more zealously did he strive to overcome the difficulties which such cases presented. When foiled in his first attempts he returned with greater eagerness to a second operation, and the case indeed was hopeless which Dieffenbach would relinquish. The largest work he has left us is upon this subject, namely, an 8vo. volume, with an atlas of plates, entitled, "Chirurgische Erfahrungen, besonders über die Wiederherstellung Zerstörter Thiele des menschlichen Körpers nach Neuen Method," 1829. He contributed also several papers to the periodical press.

As a clinical teacher, he was much sought after, and listened to with eager and profound attention by students of every nation. It was in his *clinic*, indeed, that he was seen to most advantage, which consisted, according to the German plan, in interrogating his pupils, instructing them practically in the steps of an operation, and often making them perform it before the class, under his superintendence. He was on all occasions the students' friend, and their sense of his friendship, which was evinced during his life by frequent tributes of respect shown to their admired teacher, was testified in a gratifying manner by the crowds who followed his remains to the tomb.

In the accounts given of Dieffenbach's death, apoplexy is said to have been the fatal disease; but the very sudden manner in which death occurred, renders this improbable, and we regret to see no account of a *post-mortem* examination having been made.

Such is a slight sketch of the career of two distinguished surgeons; both the sons of divines; both university professors; both distinguished as the most skilful operators of the day; both largely entrusted with the public confidence in populous capital cities; both surrounded by all the honours and emoluments their respective countries bestow; and both dying at the comparatively early age of fifty-three. So far there is a very singular coincidence in their respective careers. Did we draw a contrast between them, we should be inclined to say that Dieffenbach possessed a more inventive genius, had greater perseverance, and was more distinguished as a professor than his English rival. Liston, on the other hand, far surpassed the German professor in manual dexterity, boldness of execution, and as a bedside practitioner. Both undoubtedly were great surgeons, and have exercised a most important influence on the art to which they directed their energies and talents.—*Monthly Journal of Medical Science.*

